# The Impact of Thought Suppression on Cyberchondria

**DOI:** 10.1192/j.eurpsy.2025.1015

**Published:** 2025-08-26

**Authors:** N. Boussaid, R. Feki, I. Gassara, H. E. Mhiri, N. Smaoui, N. Charfi, J. Ben Thabet, M. Maalej, M. Maalej, S. Omri, L. Zouari

**Affiliations:** 1 Psychiatry C, Hedi Chaker university hospital center, Sfax, Tunisia

## Abstract

**Introduction:**

Cyberchondria, defined as excessive and anxiety-driven health-related internet searching, has become an increasingly significant issue in the digital age, where easy access to online health information can contribute to heightened health anxiety. Recent studies suggest that cognitive avoidance strategies, such as thought suppression, may worsen anxiety-related behaviors, including health-related internet searching.

**Objectives:**

The objective of this study was to investigate how the tendency to suppress thoughts (thought suppression) influences the development and intensity of cyberchondria in a sample of medical students.

**Methods:**

A cross-sectional study design was employed, involving a sample of 213 medical students. The White Bear Suppression Inventory (WBSI) was used to assess participants’ tendency to suppress health-related thoughts. The Cyberchondria Severity Scale (CSS) was used to measure the extent and severity of health-related internet searching behaviors. Participants also provided information about their medical history, family medical history, and psychiatric background.

**Results:**

The sample consisted predominantly of females (74.2%), with a mean age of 22.11 ± 2 years. Among the 213 participants, 22.1% reported a personal medical history, and 20.2% had a documented history of psychiatric disorders. Regarding family medical history, 59.6% of participants reported a familial history of medical conditions, and 21.6% reported a familial history of psychiatric disorders. Additionally, 39% of participants reported that family members had been hospitalized for serious illness.

The levels of cyberchondria among participants were as follows: 36.6% of participants reported low levels of cyberchondria, 41.8% reported moderate levels, 20.7% reported high levels, and 0.9% reported very high levels of cyberchondria.

A significant positive correlation was found between WBSI scores (measuring thought suppression) and Cyberchondria Severity Scale (CSS) scores (measuring health-related internet searching) (r = 0.4, p=0.02). This suggests that medical students who engaged in higher levels of thought suppression were more likely to experience intrusive health-related thoughts and engage in heightened online health searching behaviors.

**Conclusions:**

The findings of this study suggest that there is a positive relationship between thought suppression and the intensity of cyberchondria among medical students. These results highlight the importance of addressing cognitive avoidance strategies, such as thought suppression, in the management of health anxiety. Future research could explore interventions that aim to reduce thought suppression, such as cognitive-behavioral therapy (CBT) or mindfulness-based techniques, as potential strategies for alleviating cyberchondria and its associated health anxiety.

**Disclosure of Interest:**

None Declared

